# Geometric Understanding of Local Fluctuation Distribution of Conduction Time in Lined-Up Cardiomyocyte Network in Agarose-Microfabrication Multi-Electrode Measurement Assay

**DOI:** 10.3390/mi11121105

**Published:** 2020-12-14

**Authors:** Kazufumi Sakamoto, Shota Aoki, Yuhei Tanaka, Kenji Shimoda, Yoshitsune Hondo, Kenji Yasuda

**Affiliations:** 1 Department of Pure and Applied Physics, Graduate School of Advanced Science and Engineering, Waseda University, Tokyo 169-8555, Japan; sk.060603@akane.waseda.jp (K.S.); ao100sy@ruri.waseda.jp (S.A.); rmirjaa5613@fuji.waseda.jp (Y.T.); kenji.s@toki.waseda.jp (K.S.); hondoh@asagi.waseda.jp (Y.H.); 2 Department of Physics, School of Advanced Science and Engineering, Waseda University, Tokyo 169-8555, Japan

**Keywords:** on-chip cell network assay, multi microelectrode array, external field potential measurement, conduction distribution, cardiomyocyte network

## Abstract

We examined characteristics of the propagation of conduction in width-controlled cardiomyocyte cell networks for understanding the contribution of the geometrical arrangement of cardiomyocytes for their local fluctuation distribution. We tracked a series of extracellular field potentials of linearly lined-up human embryonic stem (ES) cell-derived cardiomyocytes and mouse primary cardiomyocytes with 100 kHz sampling intervals of multi-electrodes signal acquisitions and an agarose microfabrication technology to localize the cardiomyocyte geometries in the lined-up cell networks with 100–300 μm wide agarose microstructures. Conduction time between two neighbor microelectrodes (300 μm) showed Gaussian distribution. However, the distributions maintained their form regardless of its propagation distances up to 1.5 mm, meaning propagation diffusion did not occur. In contrast, when Quinidine was applied, the propagation time distributions were increased as the faster firing regulation simulation predicted. The results indicate the “faster firing regulation” is not sufficient to explain the conservation of the propagation time distribution in cardiomyocyte networks but should be expanded with a kind of community effect of cell networks, such as the lower fluctuation regulation.

## 1. Introduction

A biological cell network is composed of a group of chemically connected or functionally associated cells. In neuronal networks, a single neuron is linked to other neurons directly with elongated neurites like axons and dendrites. Hence, the conduction time was only dominated by the fast signal conduction of electrochemical impulse on the cell membrane of those neurites with more than a hundred m/s when myelinated, by crossing a synapse, where the impulse is converted from electrical to chemical and finally back to electrical [1,2,3].

In contrast to neuronal networks, propagation of firing in cardiomyocytes is thought to be a homogeneous and synchronized behavior to function the heart as blood pumping. However, when this synchronization is broken, heart function was damaged, such as ventricular arrhythmia.

Coordinated synchronous behavior of electrical conduction among cells was explained as the faster firing regulation of heart beating [4] or is called “overdrive suppression” [5]. This conduction regulation mechanism can also suppress the spontaneous beating of cells, such as Purkinje fibers, to follow the contraction impulse from the upstream sinoatrial node (SA node) with its faster beating intervals.

Massive mathematical models have also been proposed to investigate the mechanism of their beatings. One approach is an elaborated mathematical model composed of a large number of equations, each of which is reflecting the complex electrophysiological processes causing cardiomyocyte beating [6]. Another approach is a simple mathematical model using just a few ordinary equations, which are representing the key phenomenon of the membrane currents and action potentials [7,8], just the same as the famous Hodgkin-Huxley model, the FitzHugh-Nagumo model, and the Van der Pol model.

For investigating the statistical behavior of the beating and synchronization period of cardiomyocytes, we should remark the essence of beating intervals and their synchronization period from the idea that the cardiomyocytes as oscillators. From this viewpoint, starting from the phase model is one of the suitable ways for the construction of the beating interval model [9,10,11]. However, to capture the characteristic features of cardiomyocyte beating, we have to consider and incorporate the conventional stochastic phase models with three essential ideas: irreversible at firing, a refractory period after firing, and induced pulsation associated with the firing of surrounding cells. A part of those ideas was considered in the well-known integrate-and-fire model as a spiking neuron model [12,13,14].

Recently, as a practical application, fluctuation measurement of cell-to-cell conduction has been extensively examined as quasi-*in vivo* cardiotoxicity measurement assay and found that such conduction measurement can give us a more precise prediction of cardiotoxicity than the conventional in vitro screening assays, like human Ether-a-go-go Related Gene (hERG). assay [15,16,17,18]. Although those cell-to-cell conduction prediction measurements rely on the change of those fluctuations, the propagation manner of electrical conduction from perspectives of traveling distance dependency of fluctuation has not been examined well.

In this study, we examined the traveling distance dependence of conduction time fluctuation. The results showed the conduction time distribution was maintained constant regardless of the propagation distance, which was against the conventional conduction rule “overdrive suppression” only. And this conservation of conduction time distribution was disappeared when Quinidine was applied into the cardiomyocyte networks. The knowledge of length dependence of fluctuation can give us not only about the minimum requirement of cell-to-cell conduction length for reliable cardiotoxicity analysis but also about an insight into the origin of abnormality in conductance in cellular networks.

## 2. Materials and Methods

### 2.1. Embryonic Mouse Primary Cardiomyocytes

Embryonic mouse primary cardiomyocytes (primary) were isolated and purified from 14-day-old Crl:CD1(ICR) mouse embryos using a modified version of a method described in our previous reports [19,20]. All animal protocols and experiments were approved by the animal examination committee of the Institutional of Waseda University (Approval Number: 2019-A072). In brief, the embryos were rapidly removed from pregnant mice (Tokyo Laboratory Animals Science Co., Ltd., Tokyo, Japan) anesthetized with isoflurane (<2%, Wako Pure Chemical Industries, Osaka, Japan), which was volatilized by a vaporizer (Natsume Seisakusho Co., Ltd., Tokyo, Japan). The hearts of the embryos were obtained by trimming their embryos with tweezers and scissors and washed with phosphate-buffered saline (PBS, Takara Bio Inc., Shiga, Japan) containing 0.9 mM CaCl_2_ and 0.5 mM MgCl_2_ to induce heart contraction and remove corpuscles cells. The hearts were then transferred to PBS without CaCl_2_, and MgCl_2_ and the ventricles were separated from the atria, minced into 1 mm^3^ pieces with scissors. After that, they were incubated at 37 °C for 30 min in PBS containing 0.2% collagenase (Wako Pure Chemical Industries, Osaka, Japan) to digest the ventricular tissue. After this digestion step was repeated twice, the cell suspension was transferred to primary cultivation buffer (Dulbecco’s modified Eagle’s medium (DMEM, Invitrogen, Carlsbad, CA, USA) supplemented with 10% heat-inactivated fetal bovine serum (FBS, Invitrogen, Carlsbad, CA, USA), 1% penicillin-streptomycin (Invitrogen, Carlsbad, CA, USA) at 37 °C. In the subsequent experiments, the above primary cultivation medium was used to handle embryonic mouse primary cardiomyocytes. The cells were filtered through a 40 μm-nylon mesh cell strainer (BD Bioscience, Franklin Lakes, NJ, USA) to remove debris that was not able to be digested and then centrifuged at 200 g for 5 min at room temperature. After the precipitation of the cells was resuspended gently in the primary cultivation buffer, the cells were cultivated.

### 2.2. Human Embryonic Stem Cell-Derived Cardiomyocytes

Human embryonic stem cell-derived (hES) cardiomyocytes were purchased from Cellectis (hES-CMCTM002, hES cell line SA002, Gothenburg, Sweden). The hES cardiomyocytes were cultured in hES-cultivation buffer supplemented 20% heat-inactivated fetal bovine serum (Invitrogen, Carlsbad, CA, USA), 1% penicillin-streptomycin (Invitrogen, Carlsbad, CA, USA), 1% nonessential amino acids (Invitrogen, Carlsbad, CA, USA), and 0.1% β-mercaptoethanol.

### 2.3. Agarose Microfabrication

The agarose microstructures on the multi-electrode array chip (MEA chip, MED-P530A, AlphaMED scientific Co.Ltd., Osaka, Japan) were prepared as follows. First, the MEA chip was hydrophilized with a plasma ion source device (PIB-20, VACUUM DEVICE, Ibaraki, Japan). Then, the MEA chip was coated with collagen (Cellmatrix Type I-C, KP-4100, Nitta Gelatin Inc., Osaka, Japan) diluted ten times with 1 mM HCL. After drying for 30 min, the MEA chip was covered with a 0.25% agarose (Agarose Low melting point analytical grade, v2111, Promega, Madison, WI, USA) with the spin coater (1H-D7, MIKASA, Tokyo, Japan). The covered thin agarose layer was melted by the spot heating of 1480 nm focused infrared laser (RLM-1-1480, IPG Laser GmBH, Oxford, MA, USA) to form the line patterns with desired width (100, 200, and 300 μm) were fabricated.

### 2.4. Cell Culture

After the pre-cultivation of primary and hES on collagen-coated dishes for cell purification and concentration control, the cardiomyocyte networks were formed in the agarose microstructures on the MEA chips. For the pre-cultivation, 35-mm tissue culture dishes were filled with 200 μL of collagen diluted ten times with 1 mM HCL. After drying the collagen solution, the dishes were washed with the cultivation buffer. Then, 2 mL of 1.0 × 10^5^ cells/mL of cardiomyocyte suspensions were placed on the prepared collagen-coated 35-mm dishes for three days at 37 °C in 5% CO_2_. For cell collection, 1 mL of 0.25% trypsin-ethylenediaminetetraacetic acid (EDTA). were added to the dishes and incubated for 5 min at 37 °C in 5% CO_2_. Then, the solutions in the dishes were collected and centrifuged at 200 g for 5 min. After the aspiration of the supernatant, 3 mL of cultivation buffers were added as the stop solutions. After cell counting, the cells were centrifuged at 200 g for 5 min again, and the cultivation buffers were added to 3 × 10^6^ cells/mL. Ten microliters of the cell suspensions were placed to each microstructure on the MEA chips as the droplets, and the MEA chips were incubated at 37 °C for 3 h. Then, 1 mL of the cultivation buffers were added mildly and exchanged after incubating for one day. The cells were cultivated for 5–7 days, while changing the medium once every two days.

### 2.5. Measurement System

Extracellular potentials were measured using a self-made 64-channel MEA system as described before [17,18]. The MEA system was set at a sampling rate of 100 kHz with a low path filter of 2 kHz and a high path filter of 10 Hz, and signals were amplified by 5000 using an analog amplifier. The MEA chips with cardiomyocyte networks were set in the chip holder of the field potential (FP) measurement device on the stage of the inversed optical microscopy (IX-71 with an x10 phase-contrast objective lens, UPLFLN10X2PH, OLYMPUS, Tokyo, Japan) and incubated at 37 °C in 5% CO_2_.

### 2.6. Data Analysis

The conduction time (CT) is defined as the lower peak interval of the extracellular filed potential spikes measured at each electrode. A histogram of CT between each electrode in the cell network was made from 5-min measurement, and a Gaussian fitting curve was made from the obtained mean time and standard deviation of time. The short term variability (STV) of CT is the mean distance of neighboring CTs calculated as:

(1) $$STV=\sum_{i=1}^{n}\frac{|CT_{\left(i+1\right)}-CT_{i}|}{\sqrt{2}n}\times 100,$$

where $CT_{n}$ represents the CT of n-th beating.

### 2.7. Drug Administration

Dimethyl Sulfoxide (DMSO, Wako Pure Chemical Industries, Osaka, Japan) and Quinidine (Q3625-5G, Sigma-Aldrich Co. LLC., Tokyo, Japan) were purchased for drug measurements. Quinidine was dissolved in DMSO at a thousand-fold concentration to prepare stock solutions. The final concentration of Qinindine was the top critical point of its effective therapeutic concentration ($C_{M}$) in consideration of the solubility. The drug administration procedure was modified from previous experiments [15,17]. Briefly, the MEA chips of lined-up cells were selected by their beating frequency (0.6–1.1 Hz) and the waveforms of field potential (FP) recordings. The chip was placed in the holder of the on-chip MEA system and equilibrated for 5 min, and then the control FP waveforms were recorded for 10 min. Subsequently, the drug was applied to the medium at 0.1% (*v/v*) . dilution in serially increasing additions, and the FP waveforms were recorded for 10 min at each concentration. Finally, the drug-containing medium was replaced with the fresh medium after washing three times. The last 50 beats extracted from 10 min recorded FP waveform data was used for beat rate and CT (the time difference of the first peaks between separately placed electrodes in a lined-up cell-network).

### 2.8. Cell Staining

After measurement of extracellular potential of cardiomyocytes (CMs) , nuclei of CMs were stained with 4’,6-Diamidino-2-phenylindole dihydrochloride (DAPI). First, the culture medium was removed from the MEA chip. After washing the MEA chip two times with Phosphate Buffered Saline (PBS), samples were incubated with 5 μL DAPI for 60 min at room temperature. These samples were examined using fluorescence microscopy (IX71, Olympus) with a charge-coupled device (CCD) camera (ORCA-ER, C4742-80-12AG, Hamamatsu Photonics, Shizuoka, Japan), and images were obtained using AQUACOSMOS software (Hamamatsu Photonics, Shizuoka, Japan).

### 2.9. Statistical Analysis

All values are presented as mean±standard deviation (S.D.) (unless stated otherwise). All the interbeat intervals (IBIs) of cells were evaluated using the F test when comparing multiple groups. *p* < 0.05 was considered as statistically significant. F test was done by R (Ver. 4.0.3., R Core Team, R Foundation for Statistical Computing, Vienna, Austria).

### 2.10. Simulation

Mathematical model simulation was calculated with Mathematica (Ver. 12.1.1, Wolfram Research Inc., Campaign, IL, USA).

## 3. Results and Discussion

### 3.1. Distribution of Conduction Time in Width Controlled Cardiomyocyte Networks

**Figure 1:**
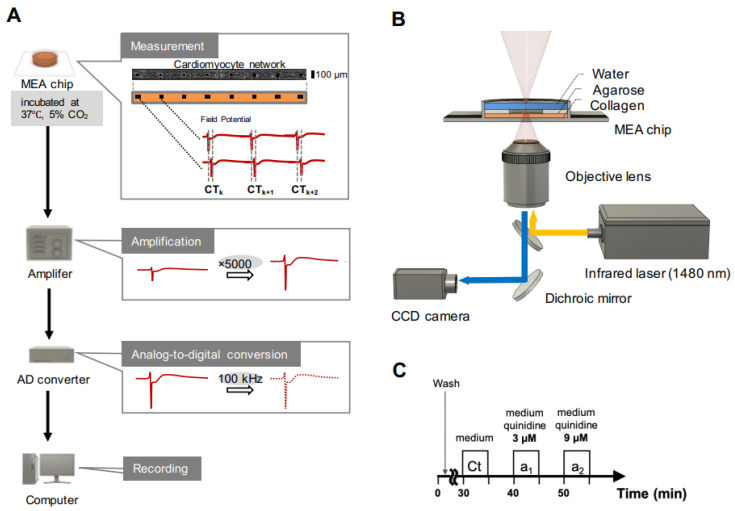
Conduction time measurement system setup and method. (**A**) Schematic drawing of multi-electrode array measurement system. Cardiomyocyte networks were cultivated in the rectangular-shaped agarose microstructures on the multi-electrode array (MEA) chip. The external field potentials (FPs) of cardiomyocytes on microelectrodes were amplified and digitally recorded to calculate the conduction time. (**B**) Setup of optical light pathways of the system. For the preparation of linearly craved MEA chip with collagen coating, a 1480-nm infrared laser beam was focused on the agarose layer of the MEA chip with x10 objective lens for spot heating of a portion of agarose layer for forming the microstructures. Phase-contrast images of cells in the microchambers were also recorded by the charge-coupled device (CCD) camera. (**C**) Time-course of drug administration. FP waveforms were recorded during 5 min of drug exposure in control medium (Ct), low concentration Quinidine (a1), and high concentration of Quinidine (a2).

**Figure 2:**
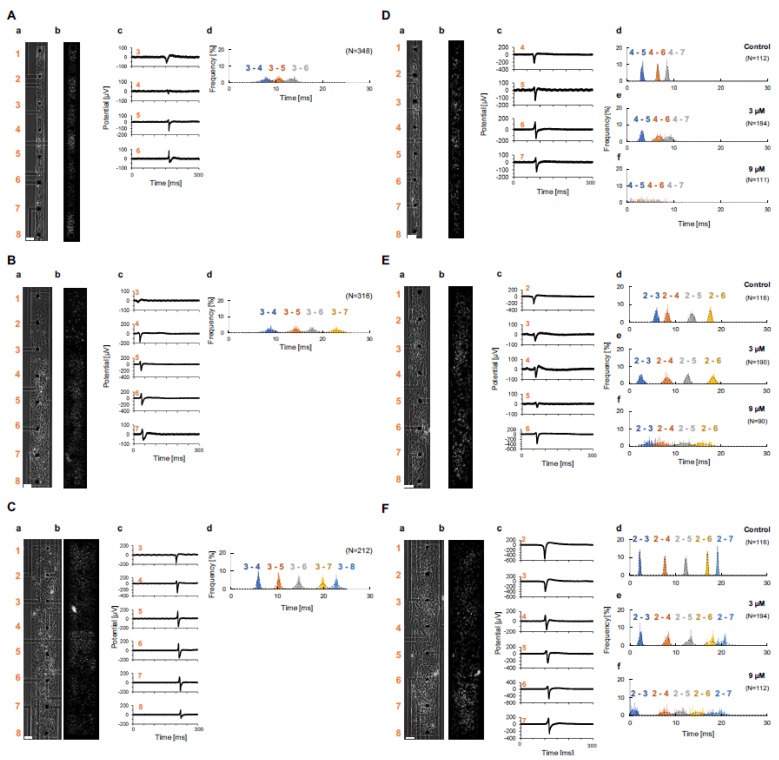
Propagation manner of linear mouse primary cardiomyocyte network and human embryonic stem cell-derived (hES) cardiomyocyte network. (**A**–**C**) Mouse primary network in 100 μm width (D), 200 μm width (E), 300 μm width (F). (a) Phase-contrast micrograph of mouse primary cardiomyocytes network with 2100 μm in length. Microelectrodes for measuring the external field potential were set 300 μm interval. The left numbers represent the associated microelectrodes. (b) Fluorescent micrograph of mouse primary cardiomyocytes in the network. The nuclei of each cell were stained by 4’,6-Diamidino-2-phenylindole dihydrochloride (DAPI). (c) Examples of Extracellular field potential signals of microelectrodes. The red numbers represent microelectrodes shown in (a). (d) Histograms of propagation time between specific two electrodes. (**D**–**F**) hES cardiomyocyte network in 100 μm width (A), 200 μm width (B), 300 μm width (C). (a) Phase contrast micrograph of hES cardiomyocyte network with 2100 μm in length. Microelectrodes for measurering the external field potential were set 300 μm interval. The left numbers represent the associated microelectrodes. (b) Fluorecent microgragh of hES cardiomyocytes in the network. The nuclei of each cells were stained by DAPI. (c) Examples of extracellular field potential signals of microelectrodes. The red numbers represent microelectrodes shown in (a). (d–f) Histgrams of propagation time between specific two electrodes; 0 μM Quinidine (Control) (d), M 3 μM Quinidine (e), and 9 μM Quinidine (f), respectively. N represents beating number. Bars, 100 μm.

We examined the characteristics of the propagation of excitation conduction in width-controlled cardiomyocyte networks. We measured the extracellular field potentials in the linearly lined-up mouse primary cardiomyocytes and human embryonic stem (hES) cell-derived cardiomyocytes with 100 kHz sampling intervals multi-electrodes signal acquisitions (Figure 1A). The width of lined-up cardiomyocyte networks was controlled geometrically by the rectangular shapes of agarose microchambers fabricated with an agarose microfabrication technology (Figure 1B). In this experiment, we constructed 100, 200, and 300 μm-width mouse primary cardiomyocyte networks (Figure 2Aa–Ca) and hES cell-derived cardiomyocyte networks (Figure 2Da–Fa). Each network was cultivated in the MEA chip for 5–7 days. Then, we obtained the waveforms of extracellular field potentials of cardiomyocytes on microelectrodes and measured the local conduction time in each network. Figure 2Ac–Fc show typical waveforms of each electrode in the control condition. The peak of the waveform was used for the timing of propagation at each electrode. Table 1 shows average conduction time and S.D. in primary cardiomyocyte networks (*n* = 2) and hES cardiomyocyte networks (*n* = 1). The conduction time of primary 1 corresponds to each histogram in Figure 2Ad–Cd, and the conduction time of hES corresponds to each histogram of control in Figure 2Dd–Fd. After the conduction measurement, we stained the cell nuclei and calculated cell density of each network. The cell density of each network was 3.0 ± 1.2 × 10^3^ cells/mm^2^ in 100 μm width, 3.5 ± 0.98 × 10^3^ cells/mm^2^ in 200 μm width, and 3.2 ± 0.76 × 10^3^ cells/mm^2^ in 300 μm width of primary network (Figure 2Ab–Cb), and 1.1 ± 0.44 × 10^3^ cells/mm^2^ in 100 μm width, 1.2 ± 0.39 × 10^3^ cells/mm^2^ in 200 μm width, and 1.3 ± 0.34 × 10^3^ cells/mm^2^ in 300 μm width hES network (Figure 2Db–Fb). These concentrations mean the averaged cell sizes were 11 μm in diameter (3.0 × 10^3^ cells/mm^2^) to 17 μm in diameter (1.3 × 10^3^ cells/mm^2^) spread two-dimensionally in the rectangular microstructures on the MEA chips. Mean number of cells in the width direction (perpendicular to the propagation direction) were 6.24 ± 0.852 cells in 100 μm width, 13.8 ± 2.63 cells in 200 μm width, and 16.6 ± 1.18 cells in 300 μm width of primary network, and 4.51 ± 0.591 cells in 100 μm width, 6.68 ± 0.480 cells in 200 μm width, and 11.2 ± 1.64 cells in 300 μm width hES network (Figure 2Db–Fb), indicating that at least four to six cells in 100 μm width chamber were lined-up perpendicular to the propagation direction and competed for propagation as the faster firing regulation.

Figure 2Ad–Fd show the distance-dependent conduction time distributions between two microelectrodes from 300 μm distance to those multiples. For example, Figure 2Ad shows the propagation from the microelectrode 3 just after the pacemaker area (between microelectrodes 2 and 3) to the microelectrode 4 (blue histogram, 300 μm distance), 3 to 5 (orange histogram, 600 μm distance), and 3 to 6 (grey histogram, 900 μm distance) in 100 μm width primary cardiomyocyte networks. As shown in the graph, the Gaussian distribution was observed even the propagation lengths were increased from 300 μm to 600 μm and 900 μm. However, there was no significant difference in variance of conduction time of 300 μm distance (Figure 2Ad3–4) and 900 μm distance (Figure 2Ad3–6) (*p* = 0.63) in 100 μm-width primary cardiomyocyte network. This tendency also appeared in 200 μm width primary cardiomyocyte network (Figure 2Bd). In addition, there was no significant difference in variance of conduction time of 300 μm distance (Figure 2Bd3–4) and 1200 μm distance (Figure 2Bd3–7) (*p* = 0.79). However, in the 300 μm width primary network (Figure 2Cd), the Gaussian distribution was spread wider when the conduction propagation distance increased from 300 μm to 1200 μm. There was difference in variance of conduction time in the 300 μm distance (Figure 2Cd3–4) and 1500 μm distance (Figure 2Cd3–8) (*p* < 0.05). In hES cardiomyocytes (Figure 2Dd,Ed,Fd), the Gaussian distribution was sharper than the mouse primary cardiomyocytes. As shown in the graph (Figure 2Dd), the Gaussian distribution was maintained similar shapes even the propagation lengths were increased to 900 μm. Similarly, these tendency appeared more clearly when the width of cardiomyocyte networks increased from 100 μm to 200 μm and 300 μm (Figure 2Ed,Fd). In all hES cardiomyocyte networks, there was no significant difference in variance of conduction time of minimum distance and max distance (300 μm distance (Figure 2Dd4–5 and 900 μm distance (Figure 2Dd4–7 (*p* = 0.76), 300 μm distance (Figure 2Ed2–3) and 1200 μm distance (Figure 2Ed2–6) (*p* = 0.32), and 300 μm distance (Figure 2Fd2–3) and 1500 μm distance (Figure 2Fd2–7) (*p* = 0.56). These differences might be caused by the difference in homogeneity and quality control of samples in primary cells and hES cells. For example, we used hES cardiomyocytes to acquire quality control and homogeneity of component cells in the networks, and the results reflected the advantage of hES cardiomyocyte purity. In contrast, in mouse primary cells, at least 10% of cells were contamination of other cells like fibroblasts, and those non-cardiomyoycyte cells might weaken the conduction efficiency of cardiomyocyte networks.

**Figure 3:**
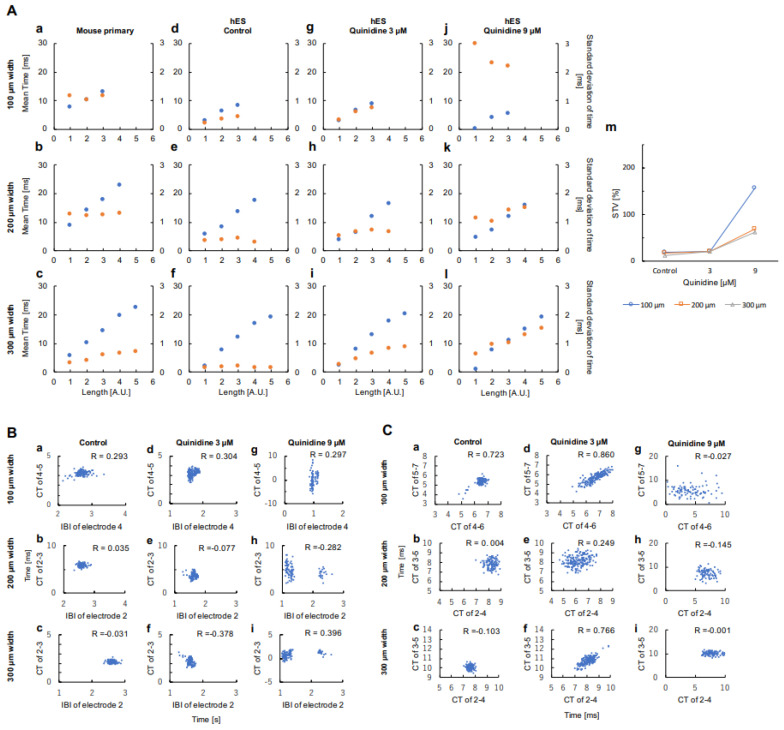
Characteristics of conduction time distribution in cardiomyocyte networks. (**A**) Comparison of fluctuations of conduction time in cardiomyocyte networks. (a)–(l) Unit length dependence of mean time (blue circles) and S.D. (orange circles) in 100 μm width (a,d,g,j), 200 μm width (b,e,h,k), and 300 μm width (c,f,i,l). Mouse primary (a–c), 0 μM Quinidine (Control) (d–f), 3 μM Quinidine (g–i), and 9 μM Quinidine (j–l). (m) The short-term variability (STV) are indicated between the neighboring electrodes from both ends of conducting section in 100 μm width (blue open circles and lines), 200 μm width (orange open squares and lines), and 300 μm width (gray open triangles and lines) were plotted. (**B**) Correlation between inter beat interval (IBI) and conduction time (CT) in 100 μm width hES network (a,d,g), 200 μm width hES network (b,e,h), 300 μm width hES network (c,f,i). The numbers represent those shown in Figure 2D–Fd–f. Zero micrometers Quinidine (Control) (a–c), 3 μM Quinidine(d–f), and 9 μM Quinidine (g–i). R represents correlation coefficient. (**C**) Cross-correlation of conduction time in 100 μm width hES network (a,d,g), 200 μm width hES network (b,e,h), 300 μm width hES network (c,f,i). The numbers represent those shown in Figure 2D–Fd–f. Zero micrometers Quinidine (Control) (a–c), 3 μM Quinidine (d–f), and 9 μM Quinidine (g–i). R represents correlation coefficient.

Conduction distance dependence of the average conduction time and standard deviation (S.D.) of primary and hES networks in 100, 200, and 300 μm width were also summarized in Figure 3Aa–f. As shown in these graphs, the mean conduction time increased in proportion to the conduction distance (a unit length d represents 300 μm, the distance of neighboring microelectrodes). In contrast, S.D. of conduction time did not diffuse (increase) or diffused slightly, while conduction unit length increased both in primary and hES (Figure 3Aa–f). These results suggest that the fluctuation of conduction time is maintained regardless of conduction unit length, or at least tends to preserve their fluctuation constant regardless of conduction distance. This tendency seems to be enhanced when the cell network width decreased in primary cardiomyocytes and increased in hES cardiomyocytes in our results. These results also indicate that the wider contaminated cell network will reduce their coordinated propagation ability, and the wider homogeneous cell network can enhance their coordinated propagation manner.

### 3.2. Effect of Fast Inward-Sodium-Current Blocking on the Distribution of Conduction Time

Next, we administered Quinidine to hES cardiomyocyte networks according to the time-course of drug administration procedure (Figure 1C). Quinidine is a class I antiarrhythmic agent (Ia) in patients with short QT syndrome or Brugada syndrome with ’use-dependent block (the block increases at higher heart rates, while the block decreases at lower heart rates)’ of voltage-gated sodium channels and is also well known for severe side effects, such as ventricular arrhythmias (the therapeutic concentration range, $C_{M}$ = 3.8 μM – 10.2 μM [21,22,23]. Hence, to control (reduce) the cell-to-cell conduction efficiency by blocking the fast inward sodium current, Quinidine was applied into the cardiomyocyte networks.

After setting to the measurement system, cells in the MEA chip were placed in the cultivation medium for 30 min (’wash’ in Figure 1C). Then, the blank medium (DMSO diluted into the cultivation medium with 0.1 % (*v/v*), ’Ct’ in Figure 1C) was applied and recorded the waveforms five min after the administration. Then, 3 μM (lowest critical point of $C_{M}$) and 9 μM (highest critical point of $C_{M}$) Quinidine were applied and recorded the waveforms for five min after each administration (’a1’ and ’a2’ in Figure 1C, respectively).

As shown in Figure 2Dd–f and Figure 3Ad,g,j, the average conduction time in 100 μm width increased with conduction distance regardless of administration concentration concentration of Quinidine. The same tendency was observed in 200 μm (Figure 2Ed–f and Figure 3Ae,h,k) and 300 μm (Figure 2Fd–f and Figure 3Af,i,l) width networks. However, the S.D. distribution was changed significantly by administration of Quinidine. While the S.D. of conduction time was constant or slightly increased in the control condition (Figure 2Dd–Fd, Figure 3Ad–f), the S.D. of conduction time of hES networks increased with increasing conduction distance in the concentration of 3 μM Quinidine (Figure 2De–Fe, Figure 3Ag–i). This tendency was observed in all three width patterns. In 9 μM Quinidine, the conduction time fluctuated further and the tendency of conduction fluctuation maintainability disappeared (Figure 2Df–Ff, Figure 3Aj–l).

In all networks, there was difference in variance of conduction time at control (Figure 2Dd–Fd) and 9 μM Quinidine (Figure 2Df–Ff); in 100 μm width network, conduction time in 900 μm distance of control (Figure 2Dd4–7) and 9 μM (Figure 2Df4–7) (*p* < 0.05); in 200 μm width network, conduction time in 1200 μm distance, control (Figure 2Ed2–6) and 9 μM (Figure 2Ef2–6) (*p* < 0.05); and in 300 μm width network, conduction time in 1500 μm distance, control (Figure 2Fd2–7) and 9 μM (Figure 2Ff2–7) (*p* < 0.05), respectively.

To check the internal correlation in those temporal fluctuations, the short-term variability (STV) between the endpoints of the conduction section was also calculated (Figure 3Am). The STVs of three width hES networks in control and 3 μM Quinidine did not show apparent differences. However, in 9 μM Quinidine, the STV increased significantly especially in 100 μm width network (138.2%, 52.2%, and 51.2% compared to control in 100 μm width, 200 μm width, and 300 μm width network, respectively). The results suggest that the ability of cell-to-cell correlation for synchronous beating decreased significantly, especially in 100 μm width in 9 μM Quinidine cell networks.

### 3.3. Correlation of Beating Intervals and Conduction Time

The relationship between interbeat intervals (IBIs) and conduction time (CT) in hES cardiomyocyte networks was also examined (Figure 3B). There was no apparent correlation between the fluctuation of IBIs and conduction times in all the width of hES cardiomyocyte network without Quinidine (Figure 3Ba–c), and also in all of the width of hES cardiomyocyte network with 3 μM Quinidine (Figure 3Bd–f). Besides, two groups of plots were observed, and there was no strong correlation between IBIs and conduction time in all the width of hES cardiomyocyte networks with 9 μM Quinidine (Figure 3Bg–i). Those results indicate that the conduction times were independent of those IBIs. Hence, the fluctuation of conduction times was not caused by the instability of IBIs. It should be noted that the IBI dependence of QT length change was well known as Bazett and Fridericia corrections [24,25]. However, the conduction time did not show such an apparent relationship with IBIs.

Two groups of IBI-dependent distributions were also observed in 200 μm and 300 μm width samples in 9 μM Quinidine (Figure 3Bh,i). The faster IBI groups’ conduction time distribution was wider than the slower groups’ distribution. It might reflect the ‘use-dependent block’ function of Quinidine. Even in the 100 μm-order conduction propagation, the faster beating cardiomyocyte networks caused more blocking of fast inward-sodium ion channels. Hence, the larger fluctuation of conduction time was observed in the faster beating cardiomyocyte networks.

### 3.4. Correlation of Conduction Time and Its Fluctuation between Neighboring Units

The relationship of conduction times among neighboring unit sections in hES cardiomyocyte network was examined (Figure 3C). In control hES cardiomyocyte network in 200– 300 μm width, there was no a significant correlation in CTs between neighboring units (Figure 3Cb–c, R = 0.004 and 0.103, respectively). However, there was an apparent correlation in 100 μm width (Figure 3Ca, R = 0.723). It is consistent with the results shown in Figure 3Ad–f, in which the distribution of their fluctuation of conduction time (S.D.) was not increased even though the conduction lengths were increased. In contrast, when 3 μM Quinidine was applied to hES cardiomyocyte networks, the correlation of conduction times between the adjacent units was appeared or significantly improved (Figure 3Cd–f, R = 0.860, 0.249, and 0.766 for 100 μm, 200 μm, and 300 μm width, respectively). The appearance of those direct correlations in conduction time among unit lengths increases fluctuation of conduction time, which means the conduction distance-dependent increase of S.D. appeared as shown in Figure 3Ag–i. It indicates that the contribution of 3 μM Quinidine was the improvement of cooperatively of conduction between neighboring units and hence the conduction length-depended conduction time fluctuation increase appeared. This result also may explain the reason why Quinidine is the class I antiarrhythmic agent in the therapeutic concentration range. Moreover, when 9 μM Quinidine was introduced to hES cardiomyocyte networks, the correlation of conduction times was dispersedly disappeared (Figure 3Cg–i, R = 0.027, 0.145 and 0.001 in 100 μm, 200 μm, and 300 μm width, respectively)). Hence, the significant increase of S.D. in Figure 3Aj–l should be caused by the disappearance of their correlations and just by the increase of individual fluctuations. The side effect of Quinidine, such as ventricular arrhythmias, also might be explained by this significant increase of S.D. in a higher concentration of Quinidine.

### 3.5. Influence of Cell Density for Conduction Velocity in Unit Length

**Figure 4:**
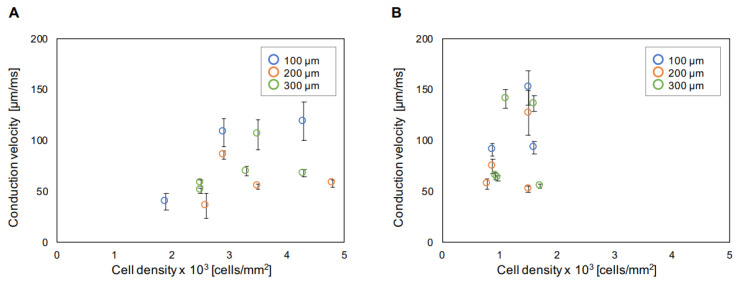
Cell concentration dependence of conduction velocity in unit lengths. Mean conduction velocities of mouse primary cells (**A**) and hES cells (**B**) in 300 μm unit length were plotted. Blue circles for 100 μm width, orange circles for 200 μm width, and green circles for 300 μm width, respectively. Error bars are S.D. of the mean conduction velocities.

In this experiment, we cultivated cardiomyocyte networks in the rectangle microchambers having different widths (Figure 2Aa–Fa). As described above, we controlled the concentration of cells 1 × 10^3^–3 × 10^3^ cells/mm^2^ to form the homogeneous monolayered two-dimensional (2D) sheet of cells in the rectangular microchambers. Figure 4 shows the cell concentration dependence of unit length conduction velocity in 100 μm, 200 μm, and 300 μm width primary (A) and hES (B) networks. Both plots showed weak correlations between cell density and conduction velocity in primary and hES cardiomyocytes (correlation coefficient = 0.39 in primary and 0.31 in hES). In both cell types, the fluctuation (S.D.) of conduction velocity was large when conduction velocity was large. In addition, when cell density and conduction velocity was lower, the conduction velocity was stable. However, in primary cardiomyocytes, the fluctuation was large when conduction velocity was small in low cell density.

### 3.6. Can the Faster Firing Regulation Explain These Conduction Characteristics?

**Figure 5:**
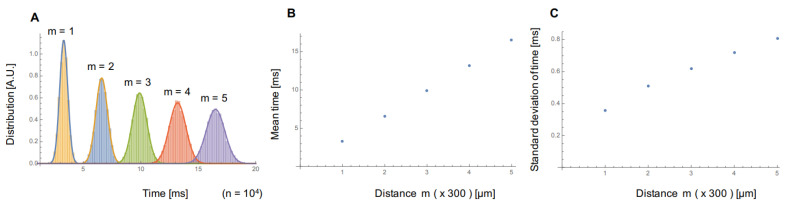
Simulation results of conduction time distribution in the faster firing regulation model. Histogram (**A**), mean value (**B**), and S.D. (**C**) of propagation time acquired from the one way random walk model ($n=10^{3}$, mean propagation time 3.3 μm/s, and S.D. 0.36 μm/s). The distribution of conduction time dispersed, and both the mean time and S.D. increased as the firing signal propagated.

When we assume the origin of cell-to-cell conduction was caused only by the faster firing regulation as a probabilistic one-way propagation model, traveling distance dependence of conduction time was increased gradually and showed the Gaussian distributions. For example, if the distribution of propagation time in the unit length (d = 300 μm) can be described as a Gaussian distribution (3.3 s as mean propagation time $\mu_{0}$, and 0.36 s as S.D. of propagation time $\sigma_{0}$ for unit length), both the mean value and S.D. of propagation time increased as the propagation distance increased. Figure 5 shows the numeric simulation of propagation time distribution for $10^{4}$ samples based on the one-way random walk model. As shown in the graphs, the propagation distance dependence of the mean value $\mu_{t}$(m) and S.D. of propagation time $\sigma_{t}$(m) are also described as:

(2) $$\mu_{t}\left(m\right)=m\mu_{0},$$

(3) $$\sigma_{t}\left(m\right)=\sqrt{m\sigma_{0}},$$

where m is the number of unit lengths for propagation, i.e., m×d is the propagation distance.

As shown in Figure 2Ad–Dd, the propagation time distribution showed similar Gaussian distributions. The mean values of conduction time in the experiments were also proportional to the distance of firing signal propagation (Figure 3Aa–f). However, the S.D. of conduction time in the experimental results showed constant or slightly increased, whereas the simulation results showed an apparent correlation between propagation distance and S.D. increase.

When we applied 3 μM Quinidine for sodium-ion channel blocking (Figure 3Ag–i), S.D. of conduction time was increased as the propagation distance increase. That might indicate that the cell-to-cell conduction of fast inward-sodium-current was not only caused by the probabilistic faster-firing regulation but also by some more cooperative contribution of cell-to-cell networks to maintain the lower constant S.D. distributions regardless of the propagation distances. In other words, it might indicate that, when the ability of fast inward-sodium-current regulation decreased, the fluctuation of conduction propagation increases.

In our previous studies, we also found that the faster firing regulation was not sufficient for understanding the synchronous behavior of spontaneously beating cardiomyocyte networks and proposed the lower fluctuation regulation rule to explain their beating synchronization tendencies [20]. The fluctuation-dissipation simulation model was proposed to explain the community effect of cardiomyocyte synchronization [26,27]. The results in Figure 3Ad–f showed that the wider the cell network width increases, the lower the fluctuation of conduction time, indicating the lower fluctuation regulation and fluctuation-dissipation model can explain a part of these experimental results. However, just the same as the faster firing model, our lower fluctuation regulation model only considered neighboring cells. It is still difficult to explain why the fluctuation was not increased during conduction.

Recently Bub’s group proposed a cellular automaton model to explain the origin of spiral formation in ventricles [28,29,30] based on the Greenberg-Hastings model [31], in which they assumed the firing of individual cells were determined by the summation of all the surrounding cells within the radius *r*. When the radius *r* was sufficiently large, that means the correlated total number of cells is sufficient, the distribution did not widen and maintained their width regardless of propagation distance. Experimental results in 3 μM Quinidine might indicate the importance of long-range communication of cells. It can be explained by reducing the radius *r* caused by blocking of sodium ion channels. If we can consider such long-range communication of surrounding cells for the decision of individual cells, we may understand those dynamics as the community effect of cell networks.

## 4. Conclusions

We reported the distance dependence of conduction time and conduction time fluctuation in linearly connected cardiomyocyte networks using agarose microstructures and the MEA device. The conservation of conduction time distribution was observed regardless of the distance in the experiments. In contrast, when Quinidine was applied to the network, the conduction distance-dependent conduction time distribution increase was observed. The origin and mechanism of this conservation phenomenon are not exact yet. However, faster firing regulation is not sufficient to explain this phenomenon.

## Figures and Tables

**Table 1:** The conduction time in cardiomyocyte networks.

			Conduction Time (Mean ± S.D.) [ms]				
			Propagation Distance [μm]				
Cell Type	Width [μm]	Sample	300	600	900	1200	1500
Mouse primary	100	1	8.11 ± 0.747	10.8 ± 0.576	13.6 ± 0.692		
		2	7.91 ± 0.599	16.7 ± 0.703	28.5 ± 0.780	39.2 ± 1.38	
	200	1	8.19 ± 1.77	13.8 ± 1.68	17.3 ± 1.75	22.6 ± 1.80	
		2	5.51 ± 0.409	9.00 ± 0.469	16.9 ± 0.603	29.4 ± 1.06	
	300	1	5.94 ± 0.256	10.4 ± 0.333	14.7 ± 0.457	19.9 ± 0.447	22.7 ± 0.473
		2	6.63 ± 0.296	12.6 ± 0.445	23.2 ± 0.915		
hES	100		3.23 ± 0.210	6.55 ± 0.193	8.54 ± 0.212		
	200		5.80 ± 0.327	8.23 ± 0.361	13.6 ± 0.422	17.6 ± 0.298	
	300		2.14 ± 0.140	7.62 ± 0.189	12.2 ± 0.212	17.0 ± 0.155	19.2 ± 0.148

Cell network widths are 100, 200, and 300 μm for two primary cardiomyocyte samples and one hES cardiomyocyte sample.

## References

[1] Brill M.H., Waxman S.G., Moore J.W., Joyner R.W. (1977). Conduction velocity and spike configuration in myelinated fibres: Computed dependence on internode distance. J. Neurol. Neurosurg. Psychiatry.

[2] Lang E.J., Rosenbluth J. (2003). Role of myelination in the development of a uniform olivocerebellar conduction time. J. Neurophysiol..

[3] Salami M., Itami C., Tsumoto T., Kimura F. (2003). Change of conduction velocity by regional myelination yields constant latency irrespective of distance between thalamus and cortex. Proc. Natl. Acad. Sci. USA.

[4] Goshima K., Tonomura Y. (1969). Synchronized beating of embryonic mouse myocardial cells mediated by FL cells in monolayer culture. Exp. Cell Res..

[5] Vassalle M. (1977). The relationship among cardiac pacemakers. Overdrive suppression. Circ. Res..

[6] Hatano A., Okada J., Washio T., Hisada T., Sugiura S. (2011). A three-dimensional simulation model of cardiomyocyte integrating excitation-contraction coupling and metabolism. Biophys. J..

[7] Keener J., Sneyd J. (1998). Mathematical Physiology.

[8] Murray J. (2002). Mathematical Biology.

[9] Kuramoto Y. (1984). Chemical Oscillations, Waves, and Turbulence.

[10] Winfree A. (2001). The Geometry of Biological Time.

[11] Kori K., Kawamura Y., Masuda N. (2012). Structure of cell networks critically determines oscillation regularity. J. Theor. Biol..

[12] Keener J.P., Hoppensteadt F.C., Rinzel J. (1981). Integrate-and-fire models of nerve membrane response to oscillatory input. SIAM J. Appl. Math..

[13] Burkitt A.N. (2006). A review of the integrate-and-fire neuron model: I. Homogeneous synaptic input. Biol. Cybern..

[14] Sacerdote L., Giraudo M.T., Bachar M., Batzel J., Ditlevsen S. (2013). Stochastic integrable and fire models: A review on mathematical methods and their applications. Stochastic Biomathematical Models with Applications to Neuronal Modeling.

[15] Kaneko T., Nomura F., Hamada T., Abe Y., Takamori H., Sakakura T., Takasuna K., Sanbuissho A., Hyllner J., Sartipy P. (2014). On-chip in vitro cell-network pre-clinical cardiac toxicity using spatiotemporal human cardiomyocyte measurement on a chip. Sci. Rep..

[16] Nozaki Y., Honda Y., Watanabe H., Saiki S., Koyabu K., Itoh T., Nagasawa C., Nakamori C., Nakayama C., Iwasaki H. (2016). CSAHi study: Validation of multi-electrode array systems (MEA60/2100) for prediction of drug-induced proarrhythmia using human iPS cell-derived cardiomyocytes-assessment of inter-facility and cells lot-to-lot-variability. Regul. Toxicol. Pharmacol..

[17] Asahi Y., Hamada T., Hattori A., Matsuura K., Odaka M., Nomura F., Kaneko T., Abe Y., Takasuna K., Sanbuissho A. (2018). On-chip spatiotemporal electrophysiological analysis of human stem cell derived cardiomyocytes enables quantitative assessment of proarrhythmia in drug development. Sci. Rep..

[18] Asahi Y., Nomura F., Abe Y., Doi M., Sakakura T., Takasuna K., Yasuda K. (2019). Electrophysiological evaluation of pentamidine and 17-AAG in human stem cell-derived cardiomyocytes for safety assessment. Eur. J. Pharmacol..

[19] Kojima K., Moriguchi H., Hattori A., Kaneko T., Yasuda K. (2003). Two-dimensional network formation of cardiac myocytes in agar microculture chip with 1480 nm infrared laser photo-thermal etching. Lab. Chip..

[20] Kojima K., Kaneko T., Yasuda K. (2006). Role of the community effect of cardiomyocyte in the entrainment and reestablishment of stable beating rhythms. Biochem. Biophys. Res. Commun..

[21] Sokolow M. (1956). Some quantitative aspects of treatment with quinidine. Ann. Intern. Med..

[22] Wu L., Guo D., Li H., Hackett J., Yan G.X., Jiao Z., Antzelevitch C., Shryock J.C., Belardinelli L. (2008). Role of late sodium current in modulating the proarrhythmic and antiarrhythmic effects of quinidine. Heart Rhythm.

[23] Paul A.A., Witchel H.J., Hancox J.C. (2002). Inhibition of the current of heterologously expressed HERG potassium channels by flecainide and comparison with quinidine, propafenone and lignocaine. Br. J. Pharmacol..

[24] Bazett H.C. (1920). An analysis of the time-relations of electrocardiograms. Heart Vessel..

[25] Fridericia L.S. (1920). Die Sytolendauer im Elektrokardiogramm bei normalen Menschen und bei Herzkranken. Acta Medica Scand..

[26] Hayashi T., Tokihiro T., Kurihara H., Yasuda K. (2017). Community effect of cardiomyocytes in beating rhythms is determined by stable cells. Sci. Rep..

[27] Hayashi T., Tokihiro T., Kurihara H., Nomura F., Yasuda K. (2018). Integrate and fire model with refractory period for synchronization of two cardiomyocytes. J. Theor. Biol..

[28] Bub G., Shrier A. (2002). Propagation through heterogeneous substrates in simple excitable media models. Chaos.

[29] Bub G., Shrier A., Glass L. (2002). Spiral wave generation in heterogeneous excitable media. Phys. Rev. Lett..

[30] Bub G., Shrier A., Glass L. (2005). Global organization of dynamics in oscillatory heterogeneous excitable media. Phys. Rev. Lett..

[31] Greenberg J.M., Hastings S.P. (1978). Spatial patterns for discrete models of diffusion in excitable media. SIAM J. Appl. Math..

